# Serosurveillance of Vaccine Preventable Diseases and Hepatitis C in Healthcare Workers from Lao PDR

**DOI:** 10.1371/journal.pone.0123647

**Published:** 2015-04-14

**Authors:** Antony P. Black, Keooudomphone Vilivong, Phonethipsavanh Nouanthong, Chanthasone Souvannaso, Judith M. Hübschen, Claude P. Muller

**Affiliations:** 1 Institute of Immunology, Luxembourg Institute of Health (former Centre de Recherche Public de la Santé)/Laboratoire National de Santé, Luxembourg, Grand-Duchy of Luxembourg; 2 Lao-Lux Laboratory, Institut Pasteur du Laos, Vientiane, Lao PDR; Public Health England, UNITED KINGDOM

## Abstract

**Background and Aims:**

Healthcare workers (HCW) have an increased risk of exposure to infectious diseases and are a potential source of infections for their patients. The Lao People’s Democratic Republic (Lao PDR) has no national policy regarding HCW vaccinations and routine vaccination coverage is low within the general population. This cross-sectional serostudy determines the level of exposure and risk of infection in Lao HCW against 6 vaccine preventable diseases and hepatitis C.

**Methods:**

1128 HCW were recruited from 3 central, 2 provincial and 8 district hospitals. Sera were tested by ELISA for the presence of antibodies and antigens to hepatitis B, hepatitis C, measles, rubella, varicella zoster, tetanus and diphtheria.

**Results:**

Only 53.1% of the HCW had protective anti-hepatitis B surface antigen antibodies (anti-HBs) with 48.8% having anti-hepatitis B core antibodies (anti-HBc), indicating previous exposure and 8.0% were hepatitis B surface antigen carriers. 3.9% were hepatitis C seropositive. Measles and rubella antibodies were detected in 95.4% and 86.2% of the HCW, with 11.9% of females being unprotected against rubella. Antibodies against varicella zoster, tetanus and diphtheria were detected in 95%, 78.8% and 55.3%, respectively. Seroprevalence varied according to age, gender and number of children.

**Conclusion:**

An unacceptably high proportion of Lao HCW remain susceptible to infection with hepatitis B, diphtheria, tetanus and rubella. Furthermore, a high number of healthcare workers are chronically infected with hepatitis B and C viruses. These data emphasize the need for a robust HCW vaccination policy in addition to increased awareness within this subpopulation.

## Introduction

Healthcare workers (HCW) have increased risk of exposure to infectious diseases and infected HCW represent a potential source for onward transmission of pathogens to susceptible patients. It is important that these risks are minimized, both by reducing exposure and by vaccination in the case of vaccine-preventable infections. In the Lao People’s Democratic Republic (PDR), the 18,017 HCW (2005), corresponded to a ratio of only 3.21 per 1000 population [1]. Whilst there are no data on the immunity of Lao HCW against important infectious diseases, exposure is probably high considering that the lack of resources undermines vaccination coverage and post-vaccination follow-up for this important risk population. In addition, low rates of childhood vaccination within the country and waning of vaccine-induced antibodies in adults further exacerbate their risk.

Hepatitis B virus (HBV) infection is endemic in Lao PDR. 45% of the general population have been exposed to HBV and 8–10% of the population are chronically infected [2–4]. HBV vaccination was first added to the Lao National Immunization Program in 2001. HCW are particularly at risk of HBV infection through contacts with their patients’ body fluids [5]. For instance, unprotected HCW have an up to 30% risk of infection in case of an injury with a HBsAg positive needle [5–7].

The mode of transmission for hepatitis C virus (HCV) is similar to HBV, although the risk of infection by HCV after a needle-stick injury is much lower (<5%) [8]. To our knowledge, there are no comprehensive seroprevalence data published on HCV in Lao PDR but about 1.1% of blood donors are HCV positive[4]. In neighbouring countries the seroprevalence is less than 2% [9,10]. There is currently no licensed vaccine against HCV.

Measles and rubella are important, highly contagious, childhood diseases which can result in fatal complications. Although they are both included in the Lao childhood national immunization programme, the epidemiology of these diseases is not well established in the country. Given the low measles vaccine coverage in Lao PDR [11] and the only recent introduction of rubella vaccine to the national immunization schedule, it is likely that a considerable proportion of adults continue to be susceptible to both measles and rubella infections. Varicella zoster virus is another important childhood infection, but the vaccine is not part of the national immunization schedule and no data exist on the disease prevalence in Lao PDR.

The diphtheria and tetanus vaccine (in combination with pertussis, haemophilus influenzae and HBV; DTP-HepB-Hib) is part of the Lao childhood vaccination schedule. However, recent diphtheria outbreaks and our own data suggest that the coverage and effectiveness of the vaccine is low [12]. There are currently no nationwide seroprevalence data available in Lao PDR on either diphtheria or tetanus.

Many countries advise that HCW should receive 3 doses of HBV vaccine [7,13–16]. Furthermore, if there is no evidence for immunity to measles, mumps and rubella, 2 doses of vaccine are recommended. Similarly, a booster for diphtheria, tetanus and pertussis and varicella vaccine should be given to non-immune HCW [16]. Other recommendations include management of risk of infection by increased awareness and protection [17]. Nevertheless, in Lao PDR there is currently no national policy with respect to immunisation or serological screening of Lao HCW.

The objective of this cross-sectional seroprevalence study is to determine the level of exposure and risk of infection in Lao HCW against HBV, HCV, measles, rubella, tetanus, diphtheria, and varicella zoster.

## Methods

### Study population

Between March and December 2013, HCW were recruited from 3 central, 2 provincial and 8 district hospitals within Vientiane Capital, Huaphan and Boulhikhamxay provinces in Lao PDR. Participation was offered to all HCW in each site and all volunteering HCW were included. Following written informed consent, 9ml of venous blood was collected and age, sex, length of time in the job, job description and numbers of children and self-reported vaccination history were recorded. As there is no vaccination programme or pre-employment serological testing for HCW in the country and no vaccination records are available in these adult age groups, we had to rely on self-reported history of vaccination. Results from the HBV and HCV serological testing were reported to the participants and anonymous data were shared with the hospital management according to the informed consent. The study was authorized by the Lao National Ethics Committee for Health Research (2013: 038/NECHR).

### Serological testing

Sera were tested by ELISA for the presence of antibodies against hepatitis B surface antigen (anti-HBs), core antigen (anti-HBc) and hepatitis C virus (anti-HCV). Anti-HBs negative, anti-HBc positive sera were also tested for the presence of hepatitis B surface antigen (HBsAg). All hepatitis ELISA kits were obtained from Diasorin, Italy. IgG antibodies specific for measles, rubella, varicella zoster, tetanus and diphtheria were detected using Euroimmun ELISA kits. According to the manufacturers’ recommendations, diphtheria antibody titres less than 0.1 IU/ml were defined as minimal or not protective, those between 0.1 and 1.0 IU/ml were considered as providing safe protection and titres above 1.0 IU/ml indicated long-term protection. Similarly, for tetanus, antibody levels less than 0.1 IU/ml were classified as negative or uncertain, those between 0.1 IU/ml and 0.5 IU/ml indicated short-term protection, between 0.5 IU/ml and 1.0 IU/ml medium protection and those above 1.0 IU/ml showed long-term protection. An ethically compliant dataset will be made available upon request, subject to approval of the competent ethical committee.

### Statistical analysis

Data were analysed using Microsoft Excel, Graphpad Prism and STATA by T tests, Chi-square and multi-variate logistical regression. Stratification of data was done to control for confounding variables.

## Results

### Participants’ profile

The 1128 participating HCW were 15 to 69 years of age (mean age 37 years); 79.8% were females. Between 51 to 71% of HCW participated in the central hospitals, 56 to 90% in the provincial hospitals and 56 to 100% in the district hospitals. The participants worked in a variety of different departments within the hospitals. The majority (64.9%) were clinical staff. More than two thirds were married (69.4%); 37.1% had no children, 17.4% had 1 child and 45.5% had 2 or more (up to 12 children). Only 262 participants provided information on the duration of hospital employment. This ranged from less than 12 months to 40 years with a mean of 13 years (Table 1).

**Table 1:** Participant profile.

**Gender**				**Number of Children**												
**Male**		**Female**		**0**					**1**						**2+**	
239 (21.2)		888 (78.2)		416 (37.1)					195 (17.4)						511 (45.5)	
**Age (years)**																
**15–24**	**25–29**		**30–34**	**35–39**		**40–44**			**45–49**			**50–54**			**55–70**	
145 (12.9)	259 (23.0)		137 (12.2)	109 (9.7)		136 (12.1)			165 (14.7)			117 (10.4)			57 (5.1)	
**Profession**																
**Lab-tech**	**Cleaner**			**Other non-clinical**			**Nurse**			**Dentist**				**Other clinical**		
79 (7.0)	59 (5.3)			253 (22.5)			500 (44.6)			36 (3.2)				195 (17.3)		
**Marital status**				**Length of time in job (years)**												
**Married**	**Un-married**			**<1**	**1–4**			**5–9**			**10–14**		**15–20**			**20–40**
781 (69.4)	344 (30.6)			17 (6.5)	84 (32.1)			30 (11.5)			13 (5.0)		19 (7.3)			99 (37.8)

Numbers represent n (%). Not all participants provided information on this aspect of the study.

### Hepatitis B and C serology

**Fig 1:**
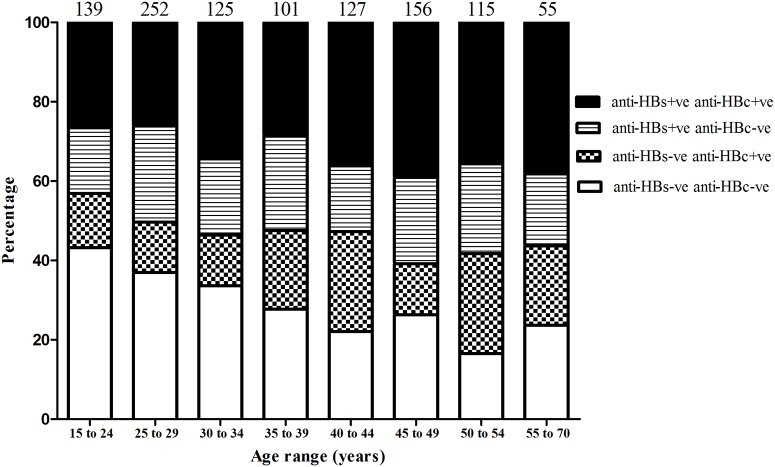
Hepatitis B seroprofile with age. Excluding 3 participants with no age data and 50 with equivocal anti-HBs data. Numbers above columns represent number per age group.

A large proportion of HCW (30.2%) were negative for both anti-HBc and anti-HBs antibodies; 16.7% were anti-HBc antibody positive, anti-HBs negative; 21.0% were anti-HBc negative, anti-HBs positive and 32.2% were anti-HBc positive, anti-HBs double-positive (Fig 1). Only 52.6% of HCW reported having been vaccinated against HBV, but among these only 61% had protective levels of anti-HBs, compared to 47.5% of individuals with uncertain vaccination history and 44.1% of those who reported having had no previous HBV vaccination (Table 2).

**Table 2:** Self-reported vaccination status versus seroprevalence and anti-HBs antibodies.

Anti-HBs antibodies	Vaccinated n (%)	Unvaccinated n (%)	Unsure n (%)	Total
Positive	336 (61.0)	166 (44.1)	57 (47.5)	559
Negative	215 (39.0)	210 (55.9)	63 (52.5)	488
Total	551	376	120	1047

Anti-HBc antibodies were detected in 59.3% of males and 46.1% of females, representing 48.8% of all participants. Below 40 years of age the anti-HBc prevalence was 42.5%, which increased to 57.6% in those 40 years or older (p<0.0001; Table 3). When stratified by age, there was no association between anti-HBc antibody prevalence and length of time in job. Out of those who reported no previous vaccination, a higher proportion were anti-HBc positive (56.1%) compared to those who reported vaccination (44.1%; p<0.001). There were no significant differences in anti-HBc prevalence between central, provincial and district hospitals.

**Table 3:** Age-stratified seroprevalence.

	Age n (%)								
	15–24	25–29	30–34	35–39	40–44	45–49	50–54	55–70	Total
**Anti-HBs+**	60 (43.2)	127 (50.4)	67 (53.6)	53 (52.5)	67 (52.8)	95 (60.9)	67 (58.3)	31 (56.4)	567 (53.0)
**HBsAg+**	6 (4.3)	16 (6.3)	5 (4.0)	13 (12.9)	18 (14.2)	9 (5.8)	13 (11.3)	6 (10.9)	86 (8.0)
**Anti-HBc+**	56 (40.3)	98 (38.9)	59 (47.2)	49 (48.5)	78 (61.4)	81 (51.9)	70 (60.9)	32 (58.2)	523 (48.8)
**Anti-HCV+**	4 (2.8)	5 (1.9)	1 (0.7)	2 (1.9)	10 (7.4)	8 (4.9)	8 (6.8)	6 (10.5)	44 (3.9)
**Anti-measles+**	125 (93.3)	233 (95.9)	123 (96.1)	95 (93.1)	121 (95.3)	152 (96.8)	107 (94.7)	55 (98.2)	1011 (95.4)
**Anti-rubella+**	109 (81.3)	194 (79.5)	120 (93.8)	88 (85.4)	113 (87.6)	143 (89.9)	99 (87.6)	53 (94.6)	919 (86.2)
**Anti-diphtheria+**	59 (43.7)	110 (44.2)	71 (55.0)	46 (44.2)	88 (67.7)	110 (69.2)	75 (64.7)	37 (66.1)	598 (55.6)
**Anti-tetanus+**	115 (84.6)	216 (86.7)	103 (81.1)	72 (69.2)	110 (84.6)	121 (76.1)	79 (68.7)	31 (55.4)	847 (78.7)
**Anti-varicella+**	127 (94.1)	228 (91.2)	122 (94.6)	102 (98.1)	126 (96.9)	155 (96.3)	113 (97.4)	54 (96.4)	1027 (95.0)

Anti-diphtheria, anti-tetanus and anti-HBs represent protective levels of antibodies.

HBsAg was detected in 11.5% of males and 7.1% of females, corresponding to 8.0% of all participants. Below 40 years of age HBsAg prevalence was 6.5%, compared to 10.2% in the older participants (p<0.05; Table 3). There was a significant difference between HBsAg prevalence in central, provincial and district hospitals (4.8%, 16.8% and 10.5%, respectively; p<0.0001) and this could not be attributed to differences in average ages (36 years old in central and 38 years old in provincial and district hospitals). There was no significant difference in HBV serology between clinical and non-clinical staff.

Anti-HCV antibodies were detected in 3.9% of participants. Those below the age of 40 years had an anti-HCV prevalence of 1.9% which increased to 6.8% in those 40 years of age or older (p<0.0001; Table 3). There was a significant difference between anti-HCV prevalence in central, provincial and district hospitals (1.7%, 6.8% and 7.4%, respectively; p<0.0001). No association was found with other measured parameters.

### Measles, rubella and varicella zoster virus serology

The majority of participants (95.4%) were positive for measles IgG, 1.7% were negative and 2.9% equivocal. Only 15.3% reported previous vaccination against measles whilst 60.9% were reported unvaccinated and 23.8% uncertain. There was no significant association between measles IgG and any of the measured parameters, including age and hospital setting.

Similarly, most participants (86.2%) were positive for rubella IgG, 10.8% negative and 3.0% equivocal. More females than males were rubella negative (11.9% and 6.6%, respectively). Out of 389 participants with no children, 15.2% remained seronegative for rubella, as compared to 8.3% of 674 participants with children (p<0.001). 13% of participants reported vaccination against rubella. There was no significant difference in rubella antibody prevalence between those reporting previous rubella vaccination and the others.

Almost all participants (95.0%) had anti-varicella IgG with 3.4% negative and 1.6% equivocal.

### Diphtheria and Tetanus serology

**Fig 2:**
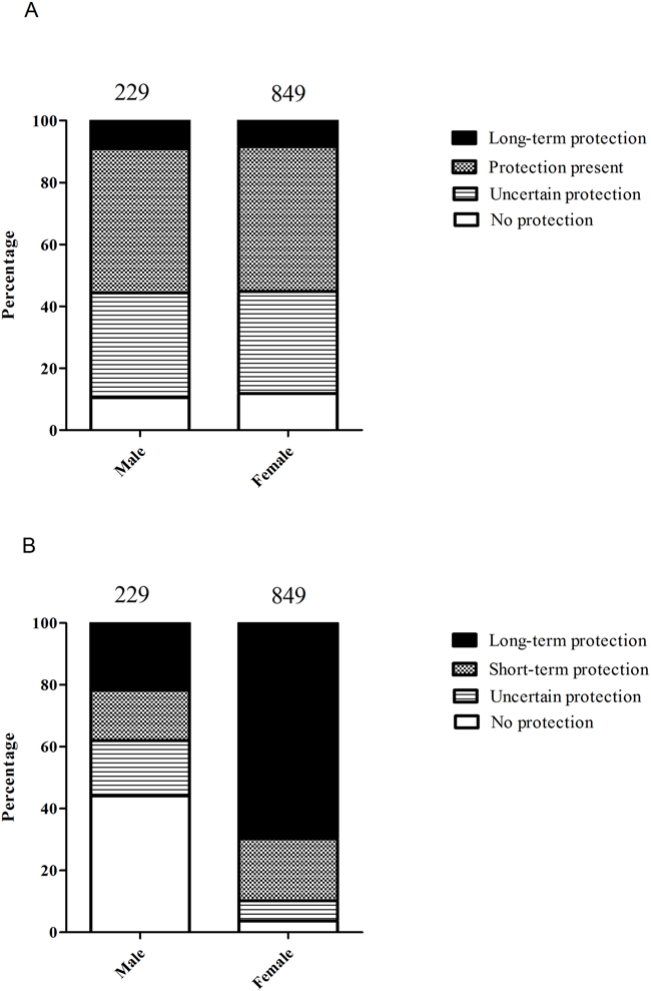
Diphteria and tetanus antibodies according to gender. A. Diphtheria antibodies according to gender. B. Tetanus antibodies according to gender. Numbers above columns represent number per age group.

**Fig 3:**
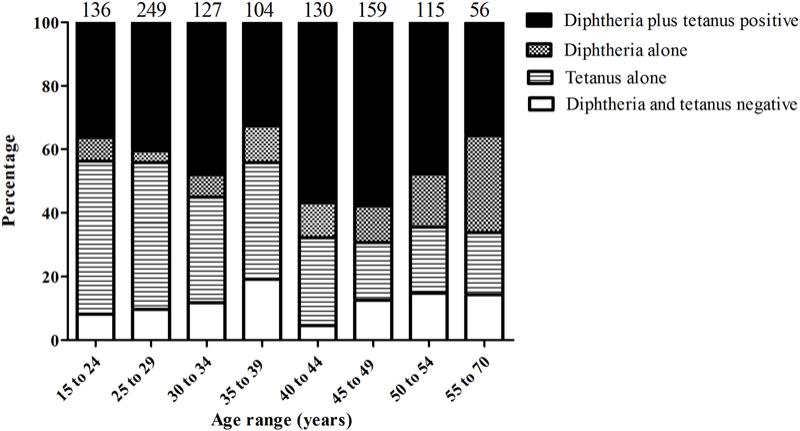
Tetanus and diphtheria antibodies according to age. Numbers above columns represent number per age group.

Protective levels of diphtheria antibodies were detected in 55.3% of participants but only 8.5% had long-term protection (Figs 2 and 3). The other 44.7% had uncertain or no protection against diphtheria. The proportion of protective diphtheria antibodies increased from 46.4% of participants younger than 40 to 67.2% of participants 40 years of age (p<0.0001; Table 3). Diphtheria antibodies alone, without tetanus antibodies, were detected in 10% of participants. There was a significant difference between anti-diphtheria prevalence in central, provincial and district hospitals (57.2%, 42.6% and 58.4%, respectively; p<0.0001). No other parameters correlated with diphtheria antibody prevalence.

Protective tetanus antibodies were detected in 78.8% of participants, with long-term protection in 59.6% and 21.2% with uncertain or no protection. There was a significant decline in tetanus antibodies with age, with 82.1% of participants under 40 years old and 74.1% aged 40 or over having protective antibodies (p<0.005; Table 3). Many more male participants had uncertain or no protection against tetanus than females (62.0% and 10.2%, respectively; p<0.0001, Fig 2). 11.2% of participants were seronegative for both diphtheria and tetanus (Fig 3).

## Discussion

In this study, we estimated the susceptibility of healthcare workers from central, provincial and district hospitals to 6 vaccine-preventable infections and against HCV. There is no national policy for HCW HBV vaccination and most hospitals do not keep records of the vaccination status of their staff. The Lao Ministry of Health has begun to provide some hospitals with adult doses of the vaccine, albeit less than required to fully vaccinate all HCW.

Childhood vaccination against HBV was only introduced gradually after 2001, i.e. well after birth of the HCW. Nevertheless, 53.1% of HCW had detectable anti-HBs antibody levels. While this is higher than the anti-HBs antibody prevalence in Lao blood donors (27.7%[3]) and pregnant Lao women (37.4%, [2]) it is very low for HCW. It reflects the poor vaccination coverage within HCW despite their high risk, and the absence of a national vaccination policy for HCW. In our study, 52.6% of participants reported previous HBV vaccination but 39% of these individuals did not have protective levels of anti-HBs antibodies. This confirms that vaccination histories are self-reported even by HCW and are poor indicators of vaccination [18–22]. Vaccination avoidance, poor quality vaccines and vaccine non-or low-responders [23] may aggravate the low anti-HBs detection rates. Importantly, given the lack of vaccination records, the proportion of HCW receiving all 3 doses of the HBV vaccine was not known in this study.

16.7% of the HCW were anti-HBc positive, anti-HBs negative including 8.0% HBsAg positive carriers. It is not clear how many of these would be protected by low levels of undetectable anti-HBs antibodies [24–26].

WHO estimates that every year in the Western Pacific Region B, which includes Lao PDR, 269,000 and 953,000 HCW are exposed to at least one percutaneous sharp injury contaminated with HCV and HBV [27]. 48.8% of our HCW had antibodies against HBc and 8.0% were HBsAg positive, similar to previous studies in adults from Lao PDR [2–4]. As in these previous studies, antibody and antigens prevalence increased significantly with age and there was no (age-independent) increase with time on the job. Interestingly, we did not see a significant difference between clinical and non-clinical staff regarding HBV exposure or HBsAg prevalence. Thus, clinical workers do not seem to have an increased risk of HBV exposure. However, the distinction between clinical and non-clinical staff is sometimes blurry, in as much as a number of administration staff had previously worked in clinical positions. However, our results may somewhat underestimate prevalences because of HBV-infected HCW who have refused to participate. A decreased prevalence of anti-HBc antibodies in those reporting previous HBV vaccination suggests some success of the immunization.

A high proportion of participants had anti-HCV antibodies (3.9%). This seroprevalence is higher than the 1.1% reported among first-time Lao blood donors [4] and also higher than neighbouring Thailand (2.15%) [9] and rural Vietnam (1%) [10]. Again, there was an increase in seroprevalence with age, indicating increased exposure in older participants but there was no clear association with clinical HCW or time on the job. The high level of chronic HBV and HCV infections may represent a considerable risk for patients, in particular during invasive procedures. That the prevalence of HBsAg and anti-HCV was significantly lower in the central hospitals than the district and provincial hospitals may reflect an increased awareness and better safety policy within those hospitals.

Diphtheria antibody seroprevalence was very low, with only 55.3% being protected. This is similar to the 60% seroprevalence in a cohort of 1000 mothers from Boulhikhamxay, Khammouane and Vientiane provinces (unpublished data). Vaccination against diphtheria, tetanus and pertussis (DTP) was introduced in Lao PDR in 1979, but nationwide coverage was only reached 10 years later. Thus, most adults older than 24 and all above 34 were born before the introduction of diphtheria containing vaccines. Nevertheless, the diphtheria seroprevalence increased with age (43.7% <25 years; 63% >35 years; p<0.0001) either because of exposure or supplementary immunization activities (probably in the general population). Diphtheria antibodies alone, without antibodies against tetanus, were detected in 10% of participants indicating past exposure to diphtheria or in some cases perhaps waning of tetanus antibodies. Since many HCW were born before general childhood vaccination against diphtheria the 45.3% of HCW with antibodies against both diphtheria and tetanus is high (36.3% of those less than 25 years of age and 48.8% of those greater than 35 years of age) and may suggest an excessively high exposure. However, we do not know how many of those may have benefited from supplementary vaccination activities. For example, the nationwide policy to reduce maternal and neonatal tetanus by tetanus toxoid vaccination of pregnant women (since 1991) and women of child-bearing age (since 2010/11) was updated from 2011 to include diphtheria containing vaccine [28] (Dr. Siddhartha Datta, personal communication). Recent diphtheria outbreaks in the country are the result of low DTP vaccine coverage (78% DTP coverage in 2010 [29]) and/or poor vaccine effectiveness [12]. Considering these outbreaks, our data emphasize the need for better vaccine coverage particularly in HCW.

The overall tetanus seroprevalence was 78.8% in our study. A significantly higher percentage of females were protected than males (89.8% versus 38.0%). We found a similarly high seroprevalence of tetanus antibodies (95%) in the mother cohort mentioned above (unpublished). This is an indication of the success of the nationwide vaccination policy for maternal and neonatal tetanus outlined above. This is also reflected in the high percentage of females who are seropositive for tetanus but not for diphtheria antibodies (38.7% of females compared to 14.4% of males). A decrease in tetanus antibody seroprevalence with age is due to older HCW that were not vaccinated as a child, during pregnancy or as women of child-bearing age. Waning antibodies may also play a role. 11.2% of participants were seronegative for both diphtheria and tetanus, indicating lack of exposure and vaccination against both diseases. Systematic (booster) vaccinations for diphtheria and tetanus should therefore be strongly recommended for Lao HCW.

The majority of participants had antibodies against measles (95.4%). As the measles vaccination was only introduced in 1989 and the vaccine coverage is still very low (68% in 2010[29]), very few of the adult HCW have ever been vaccinated, but had measles probably during early childhood. For rubella, 86.2% of participants were seropositive. As the rubella vaccination was only recently introduced, virtually all had rubella infections. While about 80% those below 25 years of age had rubella antibodies, above 45 years about 90% had such antibodies. 11.9% of the females were rubella antibody negative, 15.6% of those below <35 years and potentially at risk of a rubella infection during pregnancy. The higher seroprevalence in those with children (91.7%) compared to those without (84.8%) suggests that young children play a role in late rubella infections of women. Once again, the measles and rubella seroprevalence in the current study are similar to those found in the above cohort of mothers (approximately 91% and 89%, respectively; unpublished data). Similarly, the high seroprevalence of varicella zoster virus (95.0%) is probably a consequence of early life exposure since the vaccine is not included in the national immunization programme. These are the first data on seroprevalence of varicella zoster within the country, indicating that the virus is circulating widely within the population but the incidence of herpes zoster following reactivation of the virus is not known.

Although we do not detect increased risks of infection in clinical healthcare workers, our data show that an unacceptably high proportion of Lao HCW remain susceptible to infection with hepatitis B, diphtheria, tetanus and rubella. In Lao PDR, where serological testing is very limited even in the central hospitals and essentially impossible in most provincial and district hospitals, systemic vaccination of all HCW against HBV, diphtheria, tetanus and rubella is warranted [30]. In addition, vaccination records of HCW should be implemented.

In light of the high prevalence of chronic hepatitis B and C in HCW, risk management strategies should be enforced to protect both HCW and patients. Some authors suggest that HBsAg/ HBeAg positive or HCV RNA positive HCW should refrain from invasive procedures whilst others make no such recommendations [17,31]. Finally, the HCW attitude towards their own vaccination is likely to mirror their casual attitude to vaccination in general [32]. Therefore, vaccination awareness campaigns for HCW in hospitals would be of very broad benefit.

## References

[1] WHO. Lao People's Democratic Republic Country Health Information Profiles 2011: 162–179.

[2] Black AP, Nouanthong P, Nanthavong N, Souvannaso C, Vilivong K, Jutavijittum P, et al Hepatitis B virus in the Lao People's Democratic Republic: a cross sectional serosurvey in different cohorts. BMC Infect Dis 2014; 14: 457 10.1186/1471-2334-14-457 25149478 PMC4158128

[3] Jutavijittum P, Andernach IE, Yousukh A, Samountry B, Samountry K, Thammavong T, et al Occult hepatitis B infections among blood donors in Lao PDR. Vox Sang 2014; 106: 31–37. 10.1111/vox.12073 23931585

[4] Jutavijittum P, Yousukh A, Samountry B, Samountry K, Ounavong A, Thammavong T, et al Seroprevalence of hepatitis B and C virus infections among Lao blood donors. Southeast Asian J Trop Med Public Health 2007; 38: 674–679. 17883005

[5] Prüss-Üstün AR E. Hutin Y. Sharps injuries: global burden of disease from sharps injuries to health-care workers. WHO Environmental Burden of Disease Series 2003; 3. 10.1002/ajim.20230 16299710

[6] Sukriti Pati NT, Sethi A, Agrawal K, Agrawal K, Kumar GT, et al Low levels of awareness, vaccine coverage, and the need for boosters among health care workers in tertiary care hospitals in India. J Gastroenterol Hepatol 2008; 23: 1710–1715. 10.1111/j.1440-1746.2008.05483.x 18761556

[7] Shefer A, Atkinson W, Friedman C, Kuhar D, Mootrey G, Bialek SR, et al Immunization of Health-Care Personnel; Recommendations of the Advisory Committee on Immunization Practices (ACIP). MMWR 2011; 60. 22108587

[8] Baldo V, Floreani A, Dal Vecchio L, Cristofoletti M, Carletti M, Majori S, et al Occupational risk of blood-borne viruses in healthcare workers: a 5-year surveillance program. Infect Control Hosp Epidemiol 2002; 23: 325–327. 12083236 10.1086/502059

[9] Sunanchaikarn S, Theamboonlers A, Chongsrisawat V, Yoocharoen P, Tharmaphornpilas P, Warinsathien P, et al Seroepidemiology and genotypes of hepatitis C virus in Thailand. Asian Pac J Allergy Immunol 2007; 25: 175–182. 18035806

[10] Nguyen VT, McLaws ML, Dore GJ. Prevalence and risk factors for hepatitis C infection in rural north Vietnam. Hepatol Int 2007; 1: 387–393. 10.1007/s12072-007-9008-3 19669334 PMC2716832

[11] WHO. International review of the Expanded Programme on Immunization in the Lao People’s Democratic Republic, 5 2012.

[12] Nanthavong N, Black AP, Nouanthong P, Souvannaso C, Vilivong K, Muller CP, et al Diphtheria in Lao PDR: insufficient coverage or ineffective vaccine? Manuscript submitted. 10.1371/journal.pone.0121749 PMC4409043 25909365

[13] Sydnor E, Perl TM. Healthcare providers as sources of vaccine-preventable diseases. Vaccine 2014; 32: 4814–4822. 10.1016/j.vaccine.2014.03.097 24726251

[14] Shefer A, Strikas R, Bridges CB. Updated recommendations of the Advisory Committee on Immunization Practices for healthcare personnel vaccination: a necessary foundation for the essential work that remains to build successful programs. Infect Control Hosp Epidemiol 2012; 33: 71–74. 10.1086/662715 22173525

[15] Rose VL. ACIP releases recommendations for the immunization of health care workers. Am Fam Physician 1998; 57: 1426–1429. 9531918

[16] Kaltsas A, Sepkowitz K. Vaccinations for healthcare personnel: update on influenza, hepatitis B, and pertussis. Curr Opin Infect Dis 2013; 26: 366–377. 10.1097/QCO.0b013e3283630ee5 23806899

[17] Mele A, Ippolito G, Craxi A, Coppola RC, Petrosillo N, Piazza M, et al Risk management of HBsAg or anti-HCV positive healthcare workers in hospital. Dig Liver Dis 2001; 33: 795–802. 11838616 10.1016/s1590-8658(01)80698-8

[18] Srichomkwun P, Apisarnthanarak A, Thongphubeth K, Yuekyen C, Mundy LM. Evidence of vaccine protection among thai medical students and implications for occupational health. Infect Control Hosp Epidemiol 2009; 30: 585–588. 10.1086/597508 19419330

[19] Wiwanitkit V. How medical students in their pre-clinical year perceive their own hepatitis-B-virus status: the results of a study in a Thai medical school. Ann Trop Med Parasitol 2002; 96: 627–630. 12396325 10.1179/000349802125001672

[20] Trevisan A, Frasson C, Morandin M, Beggio M, Bruno A, Davanzo E, et al Immunity against infectious diseases: predictive value of self-reported history of vaccination and disease. Infect Control Hosp Epidemiol 2007; 28: 564–569. 17464916 10.1086/516657

[21] De Juanes JR, Gil A, San-Martin M, Gonzalez A, Esteban J, Garcia de Codes A. Seroprevalence of varicella antibodies in healthcare workers and health sciences students. Reliability of self-reported history of varicella. Vaccine 2005; 23: 1434–1436. 15670877 10.1016/j.vaccine.2004.10.003

[22] Wicker S, Allwinn R, Gottschalk R, Rabenau HF. Reliability of medical students' vaccination histories for immunisable diseases. BMC Public Health 2008; 8: 121 10.1186/1471-2458-8-121 18412957 PMC2330143

[23] Sjogren MH. Prevention of hepatitis B in nonresponders to initial hepatitis B virus vaccination. Am J Med 2005; 118 Suppl 10A: 34S–39S. 16271539 10.1016/j.amjmed.2005.07.012

[24] Yen YH, Chen CH, Wang JH, Lee CM, Changchien CS, Lu SN. Study of hepatitis B (HB) vaccine non-responsiveness among health care workers from an endemic area (Taiwan). Liver Int 2005; 25: 1162–1168. 16343067 10.1111/j.1478-3231.2005.01157.x

[25] Sunbul M, Leblebicioglu H, Esen S, Eroglu C, Barut S. Response to hepatitis B vaccine in HBsAg/anti-HBs negative and anti-HBc positive subjects. Scand J Infect Dis 2000; 32: 315–316. 10879605 10.1080/00365540050165983

[26] Kabir A, Keshvari M, Kashani AH, Alavian SM. Predicting response to HBV vaccination in people with positive anti-HBc but negative HBsAg and anti-HBs. Hum Vaccin 2008; 4: 379–383. 18398305 10.4161/hv.4.5.6010

[27] Pruss-Ustun A, Rapiti E, Hutin Y. Estimation of the global burden of disease attributable to contaminated sharps injuries among health-care workers. Am J Ind Med 2005; 48: 482–490. 16299710 10.1002/ajim.20230

[28] Comprehensive multi-year plan: 2007–2011. National Immunisation Program. Lao People's Democratic Republic. 2007.

[29] National Health Statistic Report FY 2010–2011: contributing to monitor Millenium Development Goals. 2011.

[30] Leung V, Harper S, Slavin M, Thursky K, Worth L. Are they protected? Immunity to vaccine-preventable diseases in healthcare workers at an Australian hospital. Aust N Z J Public Health 2014; 38: 83–86. 10.1111/1753-6405.12163 24494952

[31] FitzSimons D, Francois G, De Carli G, Shouval D, Pruss-Ustun A, Puro V, et al Hepatitis B virus, hepatitis C virus and other blood-borne infections in healthcare workers: guidelines for prevention and management in industrialised countries. Occup Environ Med 2008; 65: 446–451. 10.1136/oem.2006.032334 18562683

[32] Pathoumthong K, Khampanisong P, Quet F, Latthaphasavang V, Souvong V, Buisson Y. Vaccination status, knowledge and awareness towards hepatitis B among students of health professions in Vientiane, Lao PDR. Vaccine 2014; 32: 4993–4999. 10.1016/j.vaccine.2014.07.022 25066734

